# Fatal Respiratory Infections Associated with Rhinovirus Outbreak, Vietnam

**DOI:** 10.3201/eid1811.120607

**Published:** 2012-11

**Authors:** Le Thanh Hai, Vu Thi Ngoc Bich, Le Kien Ngai, Nguyen Thi Ngoc Diep, Phan Huu Phuc, Viet Pham Hung, Walter R. Taylor, Peter Horby, Nguyen Thanh Liem, Heiman F.L. Wertheim

**Affiliations:** National Hospital of Pediatrics, Hanoi, Vietnam (L.T. Hai, L.K. Ngai, P.H. Phuc, V.P. Hung, N.T. Liem);; Wellcome Trust Major Overseas Program Vietnam, Hanoi (V.T.N. Bich, N.T.N. Diep, W.R. Taylor, P. Horby, H.F.L. Wertheim);; Oxford University Clinical Research Unit, Hanoi (W.R. Taylor, P. Horby, H.F.L. Wertheim);; and University of Oxford, Oxford, UK (W.R. Taylor, P. Horby, H.F.L. Wertheim)

**Keywords:** rhinovirus, children, acute respiratory infection, outbreak, Vietnam, viruses

## Abstract

During an outbreak of severe acute respiratory infections in 2 orphanages, Vietnam, 7/12 hospitalized children died. All hospitalized children and 26/43 children from outbreak orphanages tested positive for rhinovirus versus 9/40 control children (p = 0.0005). Outbreak rhinoviruses formed a distinct genetic cluster. Human rhinovirus is an underappreciated cause of severe pneumonia in vulnerable groups.

The World Health Organization estimates that ≈2 million children die each year from acute respiratory tract infection (ARI), and most live in developing countries (*1*). Human rhinovirus (HRV), a common cause of mild upper respiratory tract infections, may also cause severe ARI in children. We report on an outbreak of severe ARI caused by HRV in children living in orphanages in Vietnam.

## The Study

During December 2007–February 2008, twelve infants <6 months of age with severe ARI infection were admitted to 2 hospitals in Hanoi. Because all 12 infants lived in 2 orphanages in Hanoi, the National Hospital of Pediatrics (NHP) initiated an outbreak investigation. Data on demographic characteristics, clinical features, and outcomes were collected for the 12 infants. Researchers visited 1 of the outbreak orphanages and 1 control orphanage (40 km apart, no severe ARI observed). Patient histories were obtained from orphanage staff; all infants were examined, and nasal and pharyngeal swab specimens were collected. Respiratory specimens were tested by bacterial culture and multiplex reverse transcription PCR (RT-PCR, Seeplex RV12 Kit; Seegene, Seoul, South Korea) for the following respiratory viruses: influenza A/B viruses, respiratory syncytial virus (RSV), rhinovirus, coronavirus (OC43/HKU1 and 229E/NL63), adenovirus, parainfluenzavirus, and human metapneumovirus (*2*). This outbreak investigation was approved by the Scientific Committee of NHP.

Twelve patients with severe ARI were admitted to the NHP intensive care unit over 38 days during the cool months of December 2007–February 2008 (temperature 5°C–14°C). The 12 hospitalized infants (11 female) were 2- to 4-months-old. Most (10/12) were from 1 orphanage, which was selected for investigation. Two hospitalized children had a known underlying condition: congenital hypothyroidism (n = 1) and HIV infection (n = 1). Seven infants were underweight (sex-specific weight-for-age; *z* score <2 SDs). All exhibited cough, coryza, wheezing, and dyspnea, and 6 (50%) had a documented fever.

Acute respiratory distress syndrome developed in all 12 a mean of 8.3 (95% CI ± 5 days) days after onset. Mean pressure of arterial oxygen/fractional inspired oxygen ratio was 66.0 (95% CI ± 30.6, range 25.1–131.0). Chest radiographs showed extensive bilateral infiltrates. Blood cultures for bacteria were negative. Despite mechanical ventilation and administration of intravenous broad-spectrum antimicrobial drugs, 7 patients died and 3 patients recovered (2 were lost to follow up). HRV was detected by RT-PCR in all infants; 2 patients were co-infected with RSV and adenovirus (Table). In addition, 1 bronchoalveolar lavage specimen from 1 patient was HRV positive.

**Table:** Detected viruses in respiratory specimens from hospitalized children, children at the outbreak orphanage, and children from the control orphanage, Hanoi, Vietnam, December 2007–February 2008*

Viral diagnosis†	Outbreak orphanage infants			Control orphanage infants
	No. (%) hospitalized, n = 12	No. (%) not hospitalized, n = 43		No. (%) not hospitalized, n = 40
Single infection				
Rhinovirus	10 (83.3)	19 (44.2)		4 (10.0)
Adenovirus	0	0		6 (15.0)
RSV	0	2 (4.7)		0
Co-infection, 2 pathogens				
Rhinovirus and adenovirus	0	2 (4.7)		4 (10.0)
Rhinovirus and RSV	0	4 (9.3)		1 (2.5)
Rhinovirus and parainfluenzavirus	0	1 (2.3)		0
Co-infection, 3 pathogens				
Rhinovirus, adenovirus, and RSV	2	0		0
Any rhinovirus	12 (100)	26 (60.5)		9 (22.5)‡

*RSV, respiratory syncytial virus. †By multiplex reverse transcription PCR (Seeplex RV12; Seegene, Seoul, South Korea). ‡χ^2^ test p = 0.0005

The outbreak orphanage was visited within 1 week of outbreak detection. The visit revealed that several other infants had been hospitalized elsewhere, but we could not obtain detailed data about them. We tested nasal–pharyngeal swab specimens (pooled) from all 43 infants (100% <12 months of age) living in the outbreak orphanage and nasal swab specimens from the 40 youngest children (97.5% <12 months of age) at the control orphanage within 2 weeks of the outbreak. In both orphanages, 5–9 children lived in 1 room and shared the same bed, wiping cloths, basic utensils such as cups, and clothing.

Most children in the outbreak orphanage were female (31/43 [72%]), compared with 50% (20/40) in the control orphanage. Of children whose specimens were tested by RT-PCR, 98% (42/43) of the infants from the outbreak orphanage had at least 1 symptom of respiratory tract infection compared with 14 (35%) of 40 infants at the control orphanage. Among children from the outbreak orphanage (both hospitalized and nonhospitalized [n = 55]), a single pathogen was identified in 31 (56.4%) infants and ≥2 pathogens were found in 9 (16.4%) children (Table). The most frequently detected pathogen was HRV (n = 38). In the control orphanage, 9 (22.5%) children tested positive for HRV, and 5 children were infected with 2 pathogens. Only nonpathogenic bacteria were cultured from the respiratory specimens (data not shown).

Because HRV was the predominant pathogen detected, we genotyped all HRV isolates directly from the specimens using a molecular typing assay based on phylogenetic comparisons of a 260-bp variable sequence (P1–P2) in the 5′-noncoding region with homologous sequences of the 101 known serotypes (*3*,*4*). We were able to sequence the P1–P2 fragment of HRV genome from 23 specimens positive for HRV: 2 were from hospitalized patients (08040R and 08043R) (Figure), 19 were from the outbreak orphanage (identification numbers starting with DA), and 5 were from the control orphanage (identification numbers starting with BaVi; Figure). The P1–P2 phylogenetic tree showed that sequences obtained from isolates from the 2 hospitalized infants were closely related to a subclade of cluster A sequences from nonhospitalized infants at the outbreak orphanage (Figure). HRVs from the control orphanage are also part of the A cluster but formed a distinct subcluster. Several children (not hospitalized) from the outbreak orphanage were also infected with HRVs from the C cluster. No children had B cluster strains.

**Figure:**
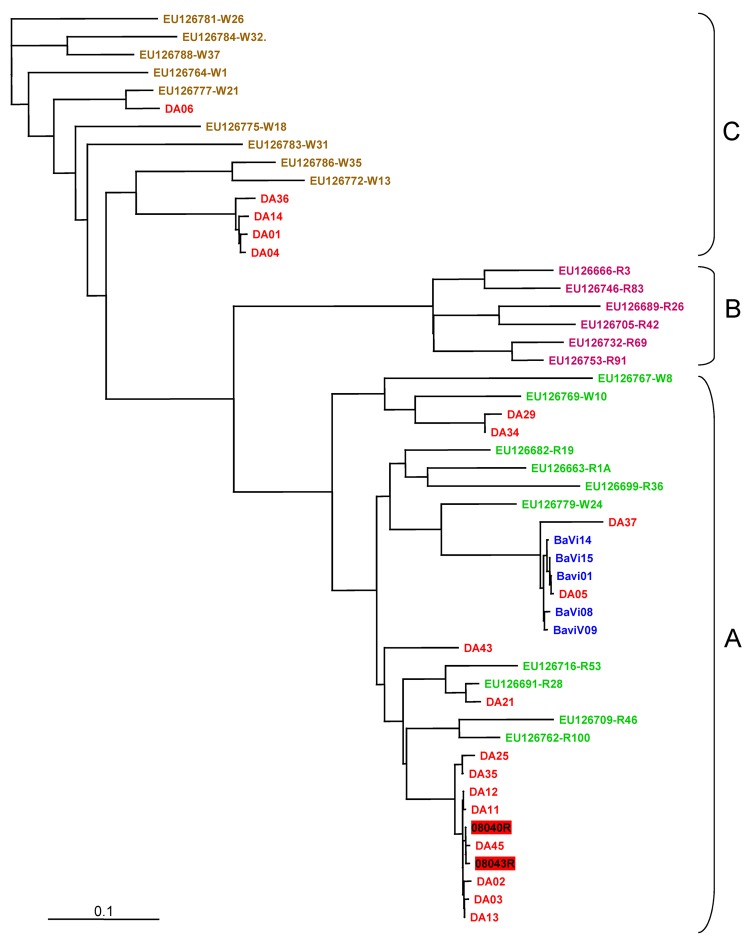
Genotyping of human rhinovirus (HRV) from outbreak and control specimens by P1–P2 sequence analysis. We compared 19 HRVs from the outbreak orphanage (red), 5 HRVs from control orphanage blue), and the following P1-P2 sequences in GenBank, according to Lee et al. (*3*). HRV-A group (green): EU126769, EU126779, EU126767, EU126699, EU126682, EU126663, EU126716, EU126691, EU126709, EU126762; HRVB group (orange): EU126732, EU126689, EU126666, EU126753, EU126705, EU126746, HRV-C group (brown): EU126786, EU126772, EU126775, EU126777, EU126783, EU126788, EU126784, EU126781, EU126764. Two HRV sequences from hospitalized patients were included (HRV number in red box). Multiple sequence alignment was performed by using the BioEdit program package (Ibis Biosciences, Carlsbad, CA, USA). Nucleotide distances were analyzed with DNAdist, the neighbor-joining tree of BioEdit package. The consensus tree was visualized by TREEVIEW v1.6.6 (Institute of Biomedical and Life Sciences, University of Glasgow, Glasgow, UK). Scale bar indicates nucleotide substitutions per site.

## Conclusions

We found that HRV was the main pathogen detected in an outbreak of severe ARI in children living in an orphanage in Vietnam. Our findings support recent studies showing that HRV may be associated with severe respiratory tract infection in infants and children (*4*–*6*). A study in central Vietnam also showed that HRV is a notable cause of ARI in children in Vietnam (*7*). Because we detected rhinovirus in all hospitalized infants, we believe that HRV was the main causal agent in this outbreak, although 2 hospitalized infants were co-infected with RSV, an unambiguous respiratory pathogen. A deep lung specimen from 1 hospitalized infant was also positive for HRV, which supports a causal relationship. Furthermore, the outbreak orphanage had significantly more HRV-positive patients than did the control orphanage, and sequence analysis showed that the outbreak isolates formed a distinct cluster, which also supports a causal role for HRV.

In addition, we found that several infants from the outbreak orphanages were infected with HRV strains belonging to the C cluster, according to P1-P2 sequence analysis. HRV-C has been associated with more severe infections and circulates worldwide (*3*,*8*–*10*). We may have missed the HRV-C strains in the clinical case-patients, because we were only able to sequence the virus from samples from 2 patients. We had to sequence directly from the specimens because we had no viral culture facility at the time of the outbreak. Phylogenetic analysis of P1–P2 sequences can distinguish HRV-B rhinoviruses, but it is limited in distinguishing HRV-A and HRV-C. Full HRV sequence analysis would be able to provide this detail, but it was not feasible for this investigation.

This outbreak investigation has some limitations. Only a small number of controls were selected from a single orphanage, and the controls were sampled later than the hospitalized patients and children from the outbreak orphanage. These limitations may have led to underdiagnosis of HRV in controls. Bacterial co-infection cannot be ruled out as a cause of more severe infection in the hospitalized infants because most were receiving antimicrobial drugs at the time respiratory specimens were collected.

Viral respiratory infections can be more severe in malnourished infants, which was likely the case for the hospitalized infants in this outbreak. In this study, co-infections (16.4%) with other respiratory viruses were detected at a similar rate as in another study in central Vietnam (12.5%) (*7*). This finding is also consistent with previous work indicating that HRV infections can occur with other respiratory viruses and lead to more severe disease (*2*,*7*). This outbreak illustrates that HRVs can cause severe pneumonia, leading to acute respiratory distress syndrome in young, vulnerable infants. HRV remains an underappreciated cause of severe pneumonia in vulnerable groups.
